# Multiplex PCR in determination of Opiinae parasitoids of fruit flies, *Bactrocera* sp., infesting star fruit and guava

**DOI:** 10.1093/jis/14.1.7

**Published:** 2014-01-01

**Authors:** S. Shariff, N. J. Ibrahim, B. M. Md-Zain, A. B. Idris, Y. Suhana, M. N. Roff, S. Yaakop

**Affiliations:** 1 School of Environmental and Natural Resource Sciences, Faculty of Science and Technology, University Kebangsaan Malaysia, 43600 Bangi, Selangor, Malaysia; 2 Horticulture Research Centre, Malaysian Agricultural Research and Development Institute (MARDI), 43400 Serdang, Selangor, Malaysia

**Keywords:** biological control programs, braconids, parasitic wasps

## Abstract

Malaysia is a tropical country that produces commercial fruits, including star fruits, *Averrhoa carambola* L. (Oxalidales: Oxalidaceae), and guavas, *Psidium guajava* L. (Myrtales: Myrtaceae). There is a high demand for these fruits, and they are planted for both local consumption and export purposes. Unfortunately, there has been a gradual reduction of these fruits, which has been shown to be related to fruit fly infestation, especially from the *Bactrocera* species. Most parasitic wasps (Hymenoptera: Braconidae: Opiinae) are known as parasitoids of fruit fly larvae. In this study, star fruits and guavas infested by fruit fry larvae were collected from the Malaysian Agricultural Research and Development Institute. The parasitized larvae were reared under laboratory conditions until the emergence of adult parasitoids. Multiplex PCR was performed to determine the braconid species using two mitochondrial DNA markers, namely cytochrome oxidase subunit I and cytochrome *b* . Two benefits of using multiplex PCR are the targeted bands can be amplified simultaneously using the same reaction and the identification process of the braconid species can be done accurately and rapidly. The species of fruit flies were confirmed using the COI marker. The results obtained from our study show that *Diachasmimorpha longicaudata* (Ashmead) (Hymenoptera: Braconidae), *Fopius arisanus* (Sonan), and *Pysttalia incisi* (Silvestri) were parasitoids associated with *Bactrocera carambolae* (Drew and Hancock) (Diptera: Tephritidae) infested star fruits. *Fopius arisanus* was also the parasitoid associated with *Bactrocera papayae* (Drew and Hancock) infested guavas. Maximum parsimony was been constructed in Opiinae species to compare tree resolution between these two genes in differentiating among closely related species. The confirmation of the relationship between braconids and fruit fly species is very important, recognized as preliminary data, and highly necessary in biological control programs.

## Introduction

Malaysia is a tropical country that cultivates and produces a high amount of star fruits, *Averrhoa carambola* L. (Oxalidales: Oxalidaceae), and guavas, *Psidium guajava* L. (Myrtales: Myrtaceae). They are the most important commercial fruits because of their high demand for exportation purposes ( Mokji and Abu Bakar 2007 ; Hii et al. 2011 ; Zakaria et al. 2011). According to the Department of Agriculture (2004) , Malaysia’s star fruits have been exported to four major market countries: the Netherlands, France, Germany, and Sin-gapore. A gradual reduction of these annual fruits has been shown to be associated with fruit fly infestation, especially from *Bactrocera* species. These species are highly diverse and broadly infest a wide variety of crops. The host records presented by Clarke et al. (2001) and Chua et al. (2010) determined that star fruits and guavas were mostly infested by *Bactrocera carambolae* (Drew and Hancock) (Diptera: Tephritidae) and *Bactrocera papayae* (Drew and Hancock), respectively. Infestation of star fruits and guavas by fruit fly species directly affects the quality of these fruits for domestic consumption and thus contributes to economic loss. Therefore, research on controlling targeted pests is valuable and significant for the maintenance and production of these fruits.

Parasitoids have been used as biological control in many countries in order to suppress the fruit fly species. Among the most commonly used biological control agents against fruit flies are members of Opiinae (Hymenoptera: Braconidae). They are known as solitary koinobiont endoparasitoids ( Wharton et al. 2000 ; Wang et al. 2003 ) because the female wasps lay their eggs in their hosts, feed on the host tissues, and emerge as adults from the host puparia. For example, *Fopius arisanus* (Sonan) (Hymenoptera: Braconidae), and *Psyttalia incisi* (Silvestri) were introduced into Hawaii and effectively reduced the oriental fruit fly, *Bactrocera dorsalis* (Hendel) (Diptera: Tephritidae) ( Harris et al. 1991 ; Vargas et al. 1993 ; Purcell 1998 ). *Bactrocera dorsalis* has become the most economically-important pest of edible fruits worldwide. Furthermore, it parasitizes the solanaceous fruit fly, *Bactrocera latifrons* (Hendel) (Diptera: Tephritidae) ( Bokonon-Ganta et al. 2007 ; McQuate et al. 2007 ), and *Bactrocera cucurbitae* (Coquillett) ( Bautista et al. 2004 ; Harris et al. 2010 ). In California, augmented releases of the parasitoid *Diachasmimorpha longicaudata* (Ashmead) (Hymenoptera: Braconidae) have been used to suppression the olive fruit fly, *Bactrocera oleae* (Rossi) ( Sime et al. 2006 ).

Several species of Opiinae have been recognized as potential parasitoids associated with *Bactrocera* species in Malaysia. The main parasitoid species observed parasitizing *B. dorsalis* complex in star fruits at Selangor and Penang orchards include *F. arisanus* , *D. longicaudata,* and *P. incisi* ( Ooi 1984 ; Vijaysegaran 1984 ; Serit et al. 1986 ; Ibrahim et al. 1995 ). According to Chua and Khoo (1995) , it was concluded that *F. arisanus* were the main parasitoid associated with *B. carambolae,* which infested star fruits. Thus, they have been used in pest management programs. Moreover, a survey conducted by Chinajariyawong et al. (2000) showed that 13 Opiinae species have been recognized as parasitoids of fruit flies from a wide variety of host plant species. However, four Opiinae species have dominated this collection, including *D. longicaudata* and *F. arisanus* . These parasitoids were determined to be associated with *B. carambolae* and *B. papayae,* which infest star fruits and guavas. All the identifications of parasitoids were based on morphological characters.

Morphological characters can sometimes be limited when it comes to differentiating between closely related parasitoid species. Therefore, molecular methods are more suitable for identification of distinguished parasitoid species ( Stouthamer et al. 1999 ; MacDonald and Loxdale 2004 ; Greenstone 2006 ; Gariepy et al. 2007 ; Bon et al. 2008 ). Accurate identifications of these parasitoids are highly significant and useful because they can be applied in biological control agents of fruit flies species ( Vargas et al. 2007 ; Harris et al. 2010 ).

Recently, multiplex PCR, has been widely used to detect parasitoid species in a single reaction. This innovative tool is particularly very time saving because of the ability to amplify different fragment sizes of DNA targets simultaneously ( Bej et al. 1991 ; Erlandson 2003 ; Traugott et al. 2006 ; Gariepy et al. 2007 ). Additionally, the sensitivity and specificity of the primers can be increased, thus preventing the formation of nonspecific products. Therefore, identification of parasitoid species can be determined accurately and rapidly. Generally, multiplex PCR has been implemented in parasitoid studies to identify multiple parasitoid species and host-parasitoid interactions. For example, Gariepy et al. (2005) developed speciesspecific PCR primers for three *Peristenus* species that parasitized *Lygus* nymphs. The results indicated that the PCR products with different amplification lengths determined the *Peristenus* species in a single reaction. Multiplex PCR has also been developed by Traugott et al. (2006) in detecting three parasitoid species of lepidopteran pests. Correct identification of these parasitoids has been used to successfully apply the parasitoids to suppress lepidopteran cabbage pests. Although this approach is widely used in applied entomology, it is also useful in the diagnosis of medical and veterinary diseases ( Xu et al. 2012 ). Due to its high sensitivity and ability to detect different DNA targets simultaneously, this approach is commonly used in pathogen diagnosis. For instance, in insect parasitology, multiplex PCR has the ability to identify different mosquito species and strains that vector malaria ( Saiwichai et al. 2007 ; Grafeo et al. 2008; Swain et al. 2009 ) and detect plant pathogens in insect vectors ( Roy et al. 2005 ; Ravikumar et al. 2011 ). These molecular applications have provided information that allows rapid and accurate identification of species.

The aim of this study was to detect and identify the parasitoid species based on the cytochrome oxidase 1 (COI) and cytochrome *b* (Cyt *b* ) gene sequences simultaneously. The benefits of using these genes are that they are relatively conserved regions and show a high degree of nucleotide variations to distinguish among the species ( Cann et al. 1984 ; Irwin et al. 1991 ; Hebert et al. 2004 ; Boehme et al. 2011 ). The COI gene is commonly known as DNA barcode and has been standardized for the molecular identification of all animals, including insects. To give better identification of these species, phylogeny has been constructed to differentiate between closely related species. Similarly, tephritid species are also confirmed by using the COI gene. Our study aimed to provide more information in recording potential parasitoids associated with fruit flies that infest star fruits and guavas. Accurate determination of these species would contribute to more effective pest management programs and directly increase production of these fruits.

## Materials and Methods

### Sample collection and DNA isolation

The larvae of tephritids that infested star fruits and guava were collected from Malaysian Agricultural Research and Development Institute (MARDI) of Kluang, Johor and Lanchang, Pahang, Malaysia ( Table 1 ). Several of these larvae were reared for adult emergence. The tephritid larvae and emerged braconids were stored in 98% ethanol for preservation. DNA was isolated from three tephritid larvae to show the replications using the standard extraction kit and protocol provided by DNeasy Blood and Tissue Kit (Qiagen, www.qiagen.com ). However, the first three steps from this kit were modified in order to develop a freezing method for the DNA isolation of adult braconid species ( Yaakop et al. 2009 ). The first step of the freezing method was adding 180 µl of buffer ATL and 20 µl of proteinase K into the samples. Then, these samples were incubated at 55° C and kept in a freezer at -22° C overnight. The steps afterwards remained the same. This method was used so that the voucher specimens would still exist and the samples for morphological re-examination would be retained.

**Table 1.**
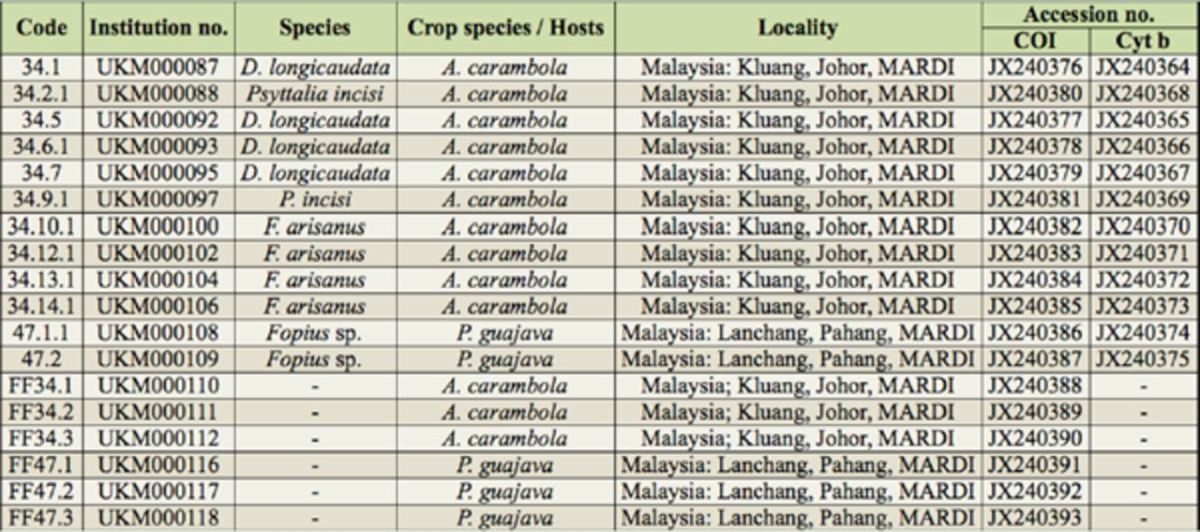
Details of braconid and tephritid samples used in analyses.

### PCR amplification

A partial mitochondrial DNA COI gene was amplified by PCR from the tephritid species using the primer designed by Han and Ro (2005) . However, for braconid species, multiplex PCR was performed using two pairs of primers designed by Folmer et al. (1994) and Simon et al. (1994) to amplify COI and Cyt *b* genes, respectively ( Table 2 ).

**Table 2.**
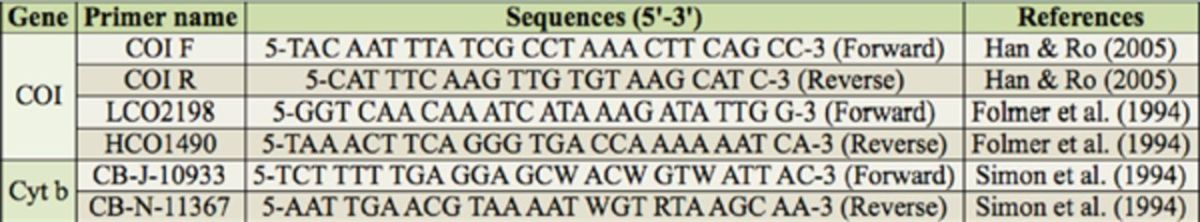
List of the primers used for PCR amplifications.

PCR was carried out using MyGene MG96G Thermalcycler (Hangzhou LongGene Scientific Instruments Co, www.longgene.com ) with the following cycles for COI of tephritids: 94° C for 3 min as initial denaturation, followed by 40 cycles of 93° C for 1 min, 56° C for 1 min, 72° C for 2 min, and the final extension step of 72° C for 15 min. For braconids, multiplex PCR was performed in order to amplify two DNA fragments simultaneously. The PCR conditions were: initial denaturation at 94° C for 3 min, followed by 40 cycles of 94° C for 1 min, 47–50° C for 1 min, and 72° C for 2 min. The final extension was at 72° C for 10 min. PCR amplification for COI tephritid was performed in a 25 µL reaction volume containing 16.50 µL dd H20, 2.5 µL PCR buffer 10X (Vivantis, www.vivantechnologies.com ), 1.30 µL 50 mM MgCl2, 0.5 µL 10 mM dNTPs, 0.5 µL foward and reverse primers (10 pmol/µL), 0.2 µL *Taq* DNA polymerase (5 U/µL) (Vivantis), and 3 µL DNA template (10–15 ng/µL). Meanwhile, for braconids, multiplex PCR was done in 50 µL reaction volume containing 33.0 µL dd H20, 5.0 µL PCR buffer 10X (Vivantis), 2.5 µL 50 mM MgCl2, 1.0 µL 10 mM dNTPs, 0.5 µL foward and reverse primers (10 pmol/µL), 0.5 µL *Taq* DNA polymerase (5 U/µL) (Vivantis), and 6 µL DNA template (1–5 ng/µL). Amplified products were separated by gel electrophoresis and purified using the Geneaid purification kit ( www.geneaid.com ). Then, they were directly sent to the sequencing service company First Base ( www.base-asia.com ) for sequencing.

### Pairwise alignment and BLAST analyses

Sequencing results from each sample were aligned using Clustal W ( Thompson et al. 1994 ). To improve the pairwise alignment results, these sequences were manually edited using BioEdit version 7.0.4 ( Hall 2005 ). Afterward, the final sequence obtained from the edited alignment was confirmed using BLAST. This approach was simple and robust for the rapid sequence comparison of query sequences to database sequences ( Altschul et al. 1990 ; Mittler et al. 2010 ). It enabled the measurement of the similarity of the sequences depending on several criteria, including expected value, maximum identical, query coverage, and maximum score, allowing the species identification of these samples to be determined.

### Phylogenetic analyses

All sequences of braconids were analyzed using PAUP 4.0b10 for phylogeny reconstruction. In PAUP analysis, maximum parsimony (MP) ( Swofford 2002 ) was performed to find the most parsimonious tree(s) with a heuristic search ( Hillis et al. 1996 ) of 1000 replications in random addition sequences and tree bisection reconnection option for branch swapping. Support for individual clades in the tree was estimated by performing bootstrap analyses with 1000 replications ( Felsenstein 1985 ). In this study, *Aspilota* sp. (GenBank Accession no: JF962946 and Z93667) was selected as the outgroup for COI and Cyt *b* genes.

## Results

### Multiplex PCR assay

**Figure 1.**
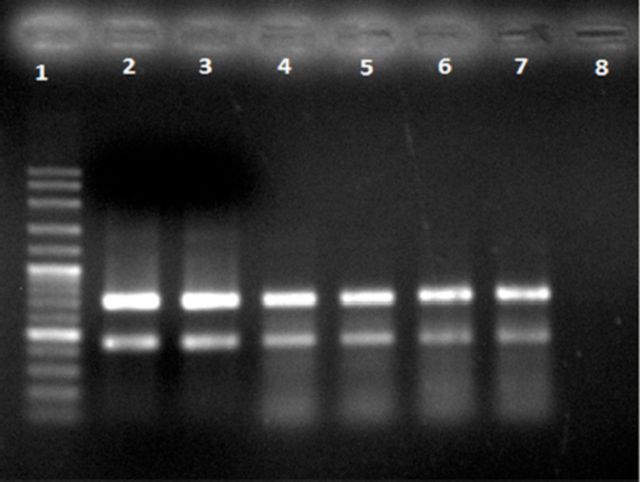
Amplifications of COI and Cyt *b* genes produced 710 bp and 440 bp of multiplex PCR products from braconid species. Lane 1: 100 bp DNA ladder; Lanes 2 and 3: *Diachasmimorpha longicaudata* (annealing temperature, Ta = 47° C); Lanes 4 and 5: *Fopius arisanus* (Ta = 50° C); Lane 6: *Psyttalia incisi* (Ta = 50° C); Lane 7: *Fopius* sp. (Ta = 50° C); Lane 8: negative control.. High quality figures are available online.

The COI and Cyt *b* genes were successfully amplified under the specific conditions described. Using the protocol described above, the COI and Cyt *b* genes produced fragments of 710 bp and 440 bp, respectively. For negative control, only chemical reagents were loaded for PCR amplification without DNA. Therefore, no PCR products were generated ( Figure 1 ). All the reagents remained constant, except for changes in annealing temperature. Optimization was done in annealing temperature to enhance the amplification of specific products. It is a crucial parameter in multiplex PCR to achieve distinct bands for each primer set ( Elizaquivel and Aznar 2008 ). The result showed each species of braconids has their own annealing temperature to produce higher amplification of COI and Cyt *b* genes simultaneously.

### Phylogenetic inference for braconids

A total of 707 bp and 438 bp sequences were obtained from multiple alignments of COI and Cyt *b* genes, respectively. Out of 707 characters from COI fragments, 162 (22.9%) were parsimony informative. However, Cyt *b* sequences were more parsimony informative, with the value of 119 (27.2%). Additionally, the conserved sites constituted 522 (73.8%) characters, showing that the COI gene was highly conserved compared to the Cyt *b* gene. The conserved sites of Cyt *b* showed 307 (70.1%) characters. The pairwise genetic distance was estimated between four braconid species based on a Kimura-two-parameter test. This algorithm distinguishes between two types of substitutions (transitions and transversions). It is useful to estimate genetic divergence ( Zhou et al. 2007 ). The highest nucleotide distance estimated from COI sequences was between *D. longicaudata* and *P. incisi* , followed by the distance between *D. longicaudata* and *F. arisanus.* Meanwhile, the highest nucleotide distance estimated from Cyt *b* sequences was between *D. longicaudata* and *Fopius* sp *.,* followed by the distance between *D. longicaudata* and *P. incisi* ( Table 3 ).

**Table 3.**
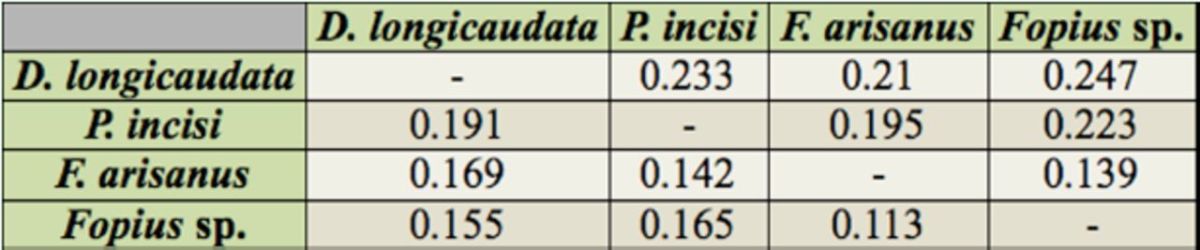
The pairwise genetic distance between the COI gene (below diagonal) and Cyt *b* gene (above diagonal) of braconid species.

**Figure 2.**
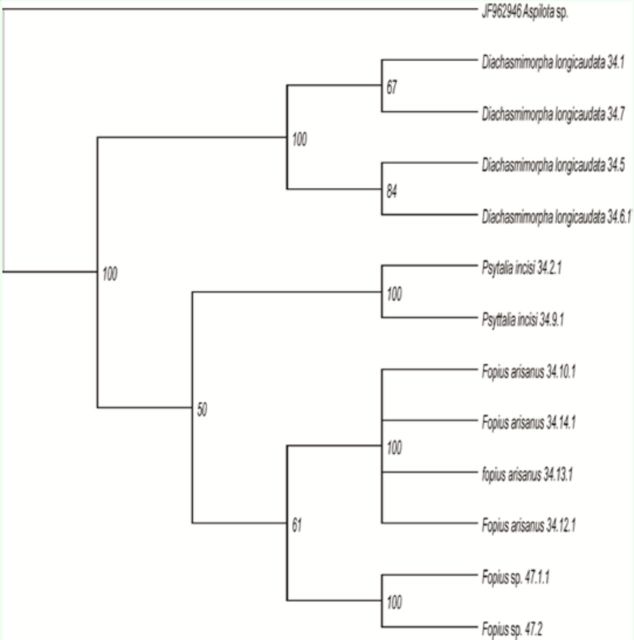
Bootstrap tree resulting from MP bootstrap analysis of COI dataset. Numbers above the branches are bootstrap values (1000 replications). High quality figures are available online.

**Figure 3.**
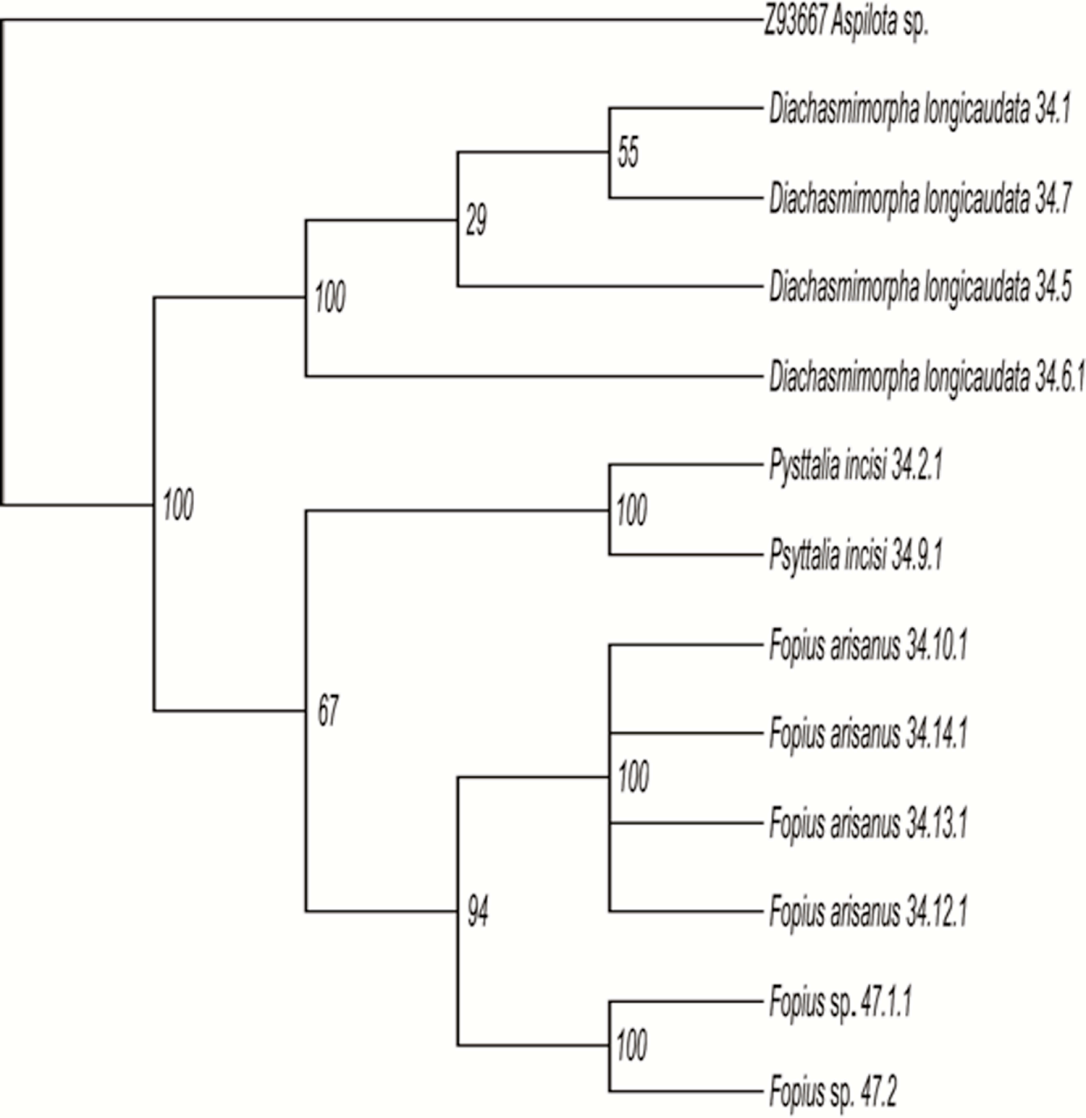
Bootstrap tree resulting from MP bootstrap analysis of Cyt *b* dataset. Numbers above the branches are bootstrap values (1000 replications). High quality figures are available online.

For the COI gene, the MP analysis based on equally-weighted characters produced two parsimonious trees ( Figure 2 ). The best tree had a minimum evolution of 269 steps, with a consistency index of 0.8104, a homoplasy index of 0.1896, and a retention index of 0.9006. However, MP analysis for the Cyt *b* gene produced three parsimonious trees with a tree length of 195 ( Figure 3 ). The best tree had a consistency index of 0.8104, a homoplasy index of 0.1896, and a retention index of 0.9006. The MP bootstrap trees produced for both genes were highly identical after bootstrap analysis with 1000 replications. Both tree topologies of MP analysis indicated *D. longicaudata* was highly distanced from other species. This species formed its own monophyletic clade, supported by a 100% bootstrap value. Otherwise, *F. arisanus* and *Fopius* sp. were indicated to be closely related species, ly related to each other.

### BLAST results of tephritids

For the tephritid species infesting star fruits, the result of BLAST showed that all three samples were referred to the *B. carambolae.* Otherwise, in guavas, the tephritid species determined from BLAST analysis were most identical to *B. papayae* . They were chosen based on higher value of maximum score, query coverage, maximum identical, and E-value (equal to zero) in BLAST analysis.

## Discussion

*Bactrocera carambolae* has been recorded as the primary pest infesting star fruits. The parasitoids that have been recorgnized based on COI and Cyt *b* genes were *D. longicaudata* , *P. incisi* , and *F. arisanus* . Previous research on star fruits also found that these Opiinae species were the main parasitoids associated with *B. dorsalis* complex, including *B. carambolae* ( Chua and Khoo 1995 ; Ibrahim et al. 1995 ; Chinajariyawong 2000 ). Otherwise, damage on guavas was caused by *B. papayae* , which is associated with the parasitoids of *Fopius* sp. A large survey conducted by Chinajariyawong et al. (2000) listed *D. longicaudata, F. arisanus* , and *P. incisi* as the most common parasitoids species detected in guavas and star fruits originating from Malaysia and Thailand. However, *Fopius vandenboschi* (Fullaway) has been recorded parasitizing *B. papayae* and *B. carambolae* in guavas. Additionally, star fruits were also the preferred host fruit for *F. arisanus* associated with *B. carambolae* ( Chua and Khoo 1995 ). Besides *F. arisanus* and *F. vandenboschi* , no record has been made regarding *Fopius* sp. attacking *Bactrocera* sp. in Malaysia. Sometimes, several parasitoid species have restricted fruit flies species in certain crops. Therefore, accurate identification of braconid or tephritid species was very significant because the species were widely used in biological control programs ( Vargas et al. 2007 : Harris et al. 2010 ).

*Diachasmimorpha longicaudata* was shown to be highly different from other species based on phylogenetic analysis, which produced the highest genetic distance between this species and others. *Fopius arisanus* and *Fopius* sp. were closely related species, with the lowest genetic distance of 0.113 and 0.139 (COI and Cyt *b* gene). The slight differences between them are due to difficulties in distinguishing them properly. This study successfully identified both species by forming two distinct clades in both topologies. To improve identification of these species, further taxonomical work should be done, especially in 47.1.1 and 47.2 individuals to determine the *Fopius* sp. This information is very important, as it can be applied to the biological control of *Fopius* sp. and *F. arisanus* tephritids, parasitoids for guavas and starfruits, respectively. Only *F. arisanus* and *F. vandenboschi* were recorded infesting star fruits and guavas in Malaysia ( Chinajariyawong et al. 2000 ). In our study, *Aspilota* sp. was selected as the outgroup because it belongs to subfamily Alysiinae, shown to be similar to the subfamily Opiinae that attacked Diptera species. However, *Aspilota* sp. were indicated parasitizing specific hosts largely from the families Phoridae and Platypezidae ( Wharton et al. 1997 ).

Therefore, detection of parasitoids species using multiplex PCR was essential in recognizing the species. Amplification of COI and Cyt *b* genes simultaneously in multiplex PCR directly gives accurate and precise information in determining species. The sensitivity of the primer also increased because two different primers were applied in same the reaction ( Traugott et al. 2006 ; Bon et al. 2008 ). Comparison information between these two markers can be made easily, and a phylogenetic tree was constructed to re-confirm these findings. Additionally, multiplex PCR considerably reduces the lengthy process of singleplex PCR ( Traugott et al. 2006 ) and is cost effective. All the samples of braconids were produced from a rearing method. Although this method required more time for braconid emergence, determination of these species was done accurately and rapidly using multiplex PCR by screening each sample with multiple target genes.

Previously, there was no record regarding utilizing COI and Cyt *b* genes simultaneously in multiplex PCR. Only the use of speciesspecific markers of COI genes was studied in detecting multi-parasitism species within their hosts ( Tilmon et al. 2000 ; Erlandson et al. 2003 ; Traugott et al. 2006 ; Bon et al. 2008 ). More interspecific variability, but moderate intraspecific variability, shown in COI genes caused it to be used often in molecular studies in Hymenoptera ( Simon et al. 1994 ; Dowton and Austin 2001 ). Furthermore, it was widely used as DNA barcoding due to the high degree of nucleotide variations for rapid identification of the species (Herbet et al. 2004; Foottit et al. 2009 ; Asokan et al. 2011 ; Boehme et al. 2011 ). These advantages are especially useful in detecting and determining immature developmental stages of the species because they often lack morphological characters. *Bactrocera carambolae* and *B. papayae* are closely related species ( Chua et al. 2010 ; Liu et al. 2011 ). The COI gene successfully differentiates between these tephritid species in larvae by showing the highest maximum identical during the BLAST analysis. On the other hand, detection of species using the Cyt *b* gene was still limited, even though this gene also showed nucleotide variations ( Irwin et al. 1991 ). Additionally, it contains both slowly and rapidly evolving codon positions, as well as more conservative regions ( Boakye et al. 1999 ; Farias et al. 2001 ; Meece et al. 2005 ). It shows that the Cyt *b* genetic differences within the insects are high enough to differentiate at the species level. However, there was no available information about Cyt *b* sequences in the GenBank regarding Opiinae parasitoids species, except studies by Gimeno et al. (1997) . Therefore, Cyt *b* sequences of *F. arisanus* , *P. incisi* , and *Fopius* sp. will be new submissions to the database. Similar topology trees produced in this study clearly resolved and indicated that these selected genes were suitable for species level classification.

### Conclusions

Multiplex PCR is an innovative tool for determining Opiinae species by amplifying multiplex targets of genes simultaneously. In our study, COI and Cyt *b* genes were selected because they showed higher nucleotide variations for rapid identification of Opiinae. This advantage would give accurate and precise information about this species and can be confirmed by constructing the phylogeny. The MP tree successfully identified and differentiated between closely related species of Opiinae. Based on the results, *D. longicaudata* , *P. incisi* , and *F. arisanus* were parasitoids associated with *B. carambolae* that infested star fruits. However, in guavas, *Fopius* sp. was the parasitoid associated with *B. papayae* . This information is useful because the braconid’s hosts were specific for certain species of fruit flies and crops. Furthermore, this information can be applied in biological control programs against fruit flies that damage star fruits and guavas.

## Abbreviations

COI: cytochrome oxidase subunit I
Cyt *b*: cytochrome *b*
MP: maximum parsimony
